# Joint attention in infants at high familial risk for autism spectrum disorder and the association with thalamic and hippocampal macrostructure

**DOI:** 10.1093/texcom/tgac029

**Published:** 2022-07-22

**Authors:** Julia T P Montenegro, Diane Seguin, Emma G Duerden

**Affiliations:** Applied Psychology, Faculty of Education, Western University, Faculty of Education Building 1137 Western Road, London, Ontario N6G1G7, Canada; Applied Psychology, Faculty of Education, Western University, Faculty of Education Building 1137 Western Road, London, Ontario N6G1G7, Canada; Physiology & Pharmacology, Schulich School of Medicine and Dentistry, Western University, Medical Science Building, Room 216 1151 Richmond St, London, Ontario N6A5C1, Canada; Applied Psychology, Faculty of Education, Western University, Faculty of Education Building 1137 Western Road, London, Ontario N6G1G7, Canada; Western Institute for Neuroscience, Western University, The Brain and Mind Institute Western Interdisciplinary Research Building, Room 3190 1151 Richmond St, London, Ontario N6A3K7, Canada; Biomedical Engineering, Faculty of Engineering, Western University, Amit Chakma Engineering Building, Room 2405 1151 Richmond St, London, Ontario N6A3K7, Canada; Psychiatry, Schulich School of Medicine and Dentistry, University of Western Ontario, Parkwood Institute Mental Health Care Building, F4-430, London, Ontario N6C0A7, Canada

**Keywords:** social gaze, MRI, infants, brain development, autism

## Abstract

Autism spectrum disorder (ASD) is a heritable neurodevelopmental disorder. Infants diagnosed with ASD can show impairments in spontaneous gaze-following and will seldom engage in joint attention (JA). The ability to initiate JA (IJA) can be more significantly impaired than the ability to respond to JA (RJA). In a longitudinal study, 101 infants who had a familial risk for ASD were enrolled (62% males). Participants completed magnetic resonance imaging scans at 4 or 6 months of age. Subcortical volumes (thalamus, hippocampus, amygdala, basal ganglia, ventral diencephalon, and cerebellum) were automatically extracted. Early gaze and JA behaviors were assessed with standardized measures. The majority of infants were IJA nonresponders (*n* = 93, 92%), and over half were RJA nonresponders (*n* = 50, 52%). In the nonresponder groups, models testing the association of subcortical volumes with later ASD diagnosis accounted for age, sex, and cerebral volumes. In the nonresponder IJA group, using regression method, the left hippocampus (*B* = −0.009, aOR = 0.991, *P* = 0.025), the right thalamus (*B* = −0.016, aOR = 0.984, *P* = 0.026), as well as the left thalamus (*B* = 0.015, aOR = 1.015, *P* = 0.019), predicted later ASD diagnosis. Alterations in thalamic and hippocampal macrostructure in at-risk infants who do not engage in IJA may reflect an enhanced vulnerability and may be the key predictors of later ASD development.

## Introduction

Autism spectrum disorder (ASD) is a neurodevelopmental disorder which includes deficits in social communication and social interactions and restricted and repetitive behaviors (American Psychiatric Association 2013). In Canada, ASD is estimated to affect 1 in 66 children and youth, or 1.5% of the child population (Ofner et al. 2018). ASD diagnosis is male-biased, with 1 in 42 males affected and with 1 in 165 females affected (Ofner et al. 2018). Additionally, there is an increased prevalence of 19% to develop ASD if the child has an older biological sibling with an ASD diagnosis (Ozonoff et al. 2011). Usually, the diagnosis of ASD is not made until the child is at least 3 years of age when communication delays are more easily identified (Zwaigenbaum 2010; Di Giorgio et al. 2016). The full manifestation of symptoms is expected to appear later in development, especially when social communication demands exceed the individual’s abilities (American Psychiatric Association 2013). Identification of the early signs of ASD during the first year of life is needed for infants to access early therapies, which can promote better social and behavioral outcomes (Gaffrey et al. 2020).

Social gaze, or direct eye contact, is an important communication channel and plays a key role in social interactions (Guellai et al. 2020). The eyes provide very subtle and complex information, which contributes to social living (Haith et al. 1977; Emery 2000). Understanding these subtle visual signals relies on the ability of the individual to correctly interpret them (Emery 2000) and this ability begins very early in development. Several studies have demonstrated that infants, from the first days after birth, perceive the gaze of others and prefer faces that engage in mutual gaze (Farroni et al. 2002; Guellai et al. 2020). Social gaze lays the foundation for developing more complex forms of social cognition, such as following the gaze of others, regulating turn-taking in conversations, and inferring others’ mental states (Emery 2000; Guellai et al. 2020). The ability to follow gaze is necessary for the development of joint attention (JA).

JA is the ability to look into the eyes and follow the gaze of others integrating a third element to the dyadic relationship with a partner (Dawson et al. 2004). JA is characterized by the alternated and coordinated attention with the partner and the object, a triadic interaction (Bruinsma et al. 2004; Tomasello et al. 2005). JA allows infants to be able to gaze into another individual’s eyes to get information about the other individual’s emotional state and about where the eye gaze is being directed. In turn, JA is essential for social emotional development and for later language acquisition (Mundy and Crowson 1997). The consistent practice of JA provides experiences for infants to build the neural systems necessary for social interactions and mentalizing (Mundy 2018).

The ability to follow gaze cues of others is known as responding to joint attention (RJA) or “gaze following” (Bottema-Beutel 2016; Mundy 2018). JA also includes the ability to initiate an interaction with a partner in order to share interest about a third element, which could be an object or event (Bottema-Beutel 2016; Mundy 2018). This ability to seek interaction is known as initiating joint attention (IJA) (Gong and Shuai 2012; Swanson et al. 2013; Bottema-Beutel 2016). JA abilities typically start emerging between 2 and 4 months of age and continue to develop throughout the second year of life (Gredebäck et al. 2010; Mundy and Jarrold 2010; Mundy 2018).

An early clinical indicator in infants who later develop ASD is the impairment in directing gaze and following the eye gaze of others (D’Entremont 2000; Charman 2003; Gredebäck et al. 2010; Ibanez et al. 2013). Impairments in spontaneous gaze-following and in engaging in JA are some of the earliest indicators of later ASD development (Charman 2003; Bruinsma et al. 2004; Werner and Dawson 2005; Ibanez et al. 2013; Bottema-Beutel 2016). Impairments in IJA are considered to be more prevalent than impairments in RJA in individuals with ASD (Ibanez et al. 2013; Gangi et al. 2014; Mundy 2018). Previous studies have shown that toddlers who had a higher likelihood of developing ASD showed weaker JA skills and engagement than typically developing toddlers, and this association was even greater for those who later developed ASD (Adamson et al. 2019).

Researchers have been investigating brain areas that could be associated with ASD core symptomatology. Previous studies found that individuals diagnosed with ASD demonstrated volumetric alterations in some specific subcortical brain regions when compared to typically developing individuals. Van Rooij et al. (2018) found an association between ASD and smaller volumes of subcortical areas such as the pallidum, putamen, nucleus accumbens, and amygdala. The findings suggest that alterations in those areas could be associated with social-motivational and cognitive/motor impairments in ASD and with mechanisms of social reward differences observed in individuals with ASD (Van Rooij et al. 2018). Other studies, however, found that the amygdala was enlarged in children with ASD compared to the control group (Mosconi et al. 2009; Nordahl et al. 2012).

Research studies report that subcortical brain areas, such as the amygdala, the striatum, and hippocampus, are implicated in JA behaviors (Gordon et al. 2013; Mundy 2018). Mosconi et al. (2009) found increased amygdala volume in 2- and 4-year-old children diagnosed with ASD and also found an association between increased amygdala volumes and decreased JA ability in 4-year-old children with ASD. Sussman et al. (2015) reported smaller subcortical volumes in brain areas, such as in the hippocampus, thalamus, and globus pallidus in children and adolescents with ASD, relative to the total brain volume. Smaller thalamic volumes have also been reported in children diagnosed with ASD compared to control groups (Tamura et al. 2010; Sussman et al. 2015). Whether comparable associations between JA behaviors and subcortical volumetric development occurs in infancy is currently understudied.

Differences in subcortical volumes between ASD and non-ASD participants have been the focus behind a large volume of research. For example, amygdala enlargement has been reported in infants and children later diagnosed with ASD (Avino et al. 2018; Li et al. 2019), and differential amygdala subnuclei volumes and growth trajectories have been associated with ASD symptomatology (Seguin et al. 2021). Morphological alterations of the putamen, pallidum, and thalamus have been associated with ASD (Schuetze et al. 2016; Van Rooji et al. 2018), while findings on the relationship between hippocampal volumes and ASD symptoms have been mixed (Bigler et al. 2003; Schumann et al. 2004; Nicolson et al. 2006; Li et al. 2022). As subcortical structures have been implicated in both ASD symptomatology and the emergence of ASD, research is needed to investigate whether alterations in subcortical morphology and JA behaviors can be used to predict ASD diagnoses in early infancy.

In the current work, we examined JA abilities in young infants who carried a familial risk for the development of ASD. Infants with a sibling diagnosed with ASD were assessed for JA and underwent structural neuroimaging at 4 or 6 months of age. We examined the association between subcortical brain volumes, behavioral measures of responding to and initiating JA, and ASD diagnostic status. We hypothesized that JA behaviors will be limited or absent in infants who are later diagnosed with ASD and that subcortical volumes will predict ASD diagnosis in infants. A better understanding of early JA and the underlying neural mechanisms could identify key windows for intervention as well as biomarkers for use as identification tools for accessing early interventions in order to promote improved social and behavioral outcomes in infants who have an increased likelihood for the development of ASD (Zwaigenbaum 2010).

## Material and Methods

### Participants

Initially, data from 131 infants were collected through the National Database for Autism Research repository as part of the National Institute of Mental Health Data Archive (NDA) (Payakachat et al. 2016). Individuals were recruited as part of a longitudinal study to examine brain-based and behavioral phenotypes in infants who carry a familial risk for the development of ASD. All infants in the study had a sibling who was diagnosed with ASD. The infants were recruited from multiple sites through institutions that were part of the Autism Centers of Excellence (ACE) Program and the Infant Brain Imaging Study (IBIS) Network. The infants completed magnetic resonance imaging (MRI) scans when they were 4 or 6 months of age and completed behavioral assessments at multiple time points from 5 to 15 months of age. Data from both studies were combined using the data from all infants who had image data available at 4 and/or 6 months of age from the IBIS Network (*n* = 71) and ACE Study (*n* = 60).

Data from the Vineland Adaptive Behavior Scale-II (VABS-II) and the Autism Observation Scale for Infants (AOSI) were used to examine early gaze and JA behaviors and data from the Autism Diagnostic Observational Schedule, General (ADOS-G) (Lord et al. 2000) was used to confirm the diagnosis. Data from 24 infants were excluded as those infants did not complete the ADOS-G at any time point, leading to a sample of 107 infants (63% males). All the 107 infants had MR image data available at 4 or 6 months of age, had behavioral measures for the AOSI and the Vineland completed at some time point, and had completed the ADOS-G. For the current study, scores from the assessments completed at the closest time point to the MRI scans were used. The age of the first assessment completed for the VABS-II varied from 5 to 14 months of age and from 6 to 15 months of age for the AOSI. As JA is a developmentally sensitive process, we chose to limit the behavioral assessments that were performed at 9 months of age or below to ensure all behavioral and imaging data were collected within a comparable developmental period. The mean age for the acquisition of the MRI was 5.73 months of age. For the behavioral measures Vineland and AOSI, the mean ages were 6.62 and 6.64 months, respectively. After applying the exclusion criteria, we had a total of 101 participants (62% males) in our sample. A total of 20 infants (18.7%) in the sample received a diagnosis of ASD, which was confirmed by the ADOS-G. A total of 17 of the children diagnosed were male (85%) and 3 were female (15%). All participants who were later diagnosed as ASD had data collected prior to 9 months of age and were included in the analysis.

### Behavioral and Developmental Assessments

#### Vineland Adaptive Behavioral Scale-II

The VABS-II is a standardized norm-referenced measure of adaptive behavior (Sparrow 2011). The questionnaire assesses 4 adaptive domains: Communication, Daily Living Skills, Socialization, and Motor Skills. There is 1 additional domain, Maladaptive Behavior, which is optional to complete (Community-University Partnership for the Study of Children 2011) . The questionnaire is suitable for infants from birth to adults of 90 years of age (Sparrow 2011). The questionnaire is available as an interview form (semi-structured, open-ended interview) as well as a parent/caregiver form (Community-University Partnership for the Study of Children 2011). The interview and parent-caregiver formats do not differ from each other in terms of content but differ in how it is administrated (Community-University Partnership for the Study of Children 2011). The scores for each of the items range from 0 to 2, indicating how often the child displays the behavior (0 = never; 1 = sometimes/partially; 2 = usually).

#### Autism Observational Scale of Infants

The AOSI is a measure to detect early signs of ASD, particularly for infants who have a familial risk for ASD, to be used exclusively in research contexts (Bryson et al. 2008). The AOSI is composed of semi-structured activities administered by an expert examiner (Bryson et al. 2008). The activities are divided into 19 tasks in which the examiner observes specific signs of autism in infants (Bryson and Zwaigenbaum 2014). The AOSI was created based on the infants’ developmental trajectories (Bryson et al. 2008). Its administration is characterized by an interactive play between an infant and an examiner, while assessing infants’ target behaviors (e.g. visual tracking, disengagement of attention, orientation to name, reciprocal social smiling, differential response to facial emotion, and social anticipation and imitation) (Bryson et al. 2008). The measure can be used to assess infants from 6 to 18 months of age for ASD (Bryson et al. 2008). The scores for each of the items range from 0 to 3, indicating if the child displays typical behavior (0 = typical behavior; 1 = inconsistent/partial behavior; 2 = impaired/atypical behavior; 3 = total lack of behavior).

### MRI acquisition

All images were acquired on a Siemens 3T scanner. T1-weighted MR images were acquired with 160 sagittal slices using parameters: repetition time (TR) and echo time (TE)—TR/TE = 2,400/3.16 ms and voxel resolution = 1 × 1 × 1 mm^3^. For this study, T1-weighted images obtained in babies aged 4 or 6 months of age were selected for the subsequent image segmentation.

### MR image processing

The quality of the acquired images was visually inspected for motion and other artifacts. The T1-weighted images were subsequently analyzed using recon-all command using Infant FreeSurfer (Fischl 2012; de Macedo Rodrigues et al. 2015; Zollei et al. 2020). Infant FreeSurfer is an automatic processing stream for T1-weighted MRI scans in infants (Zollei et al. 2020). Automatic processing steps include intensity normalization, skull stripping, and segmentation of the cortex, white matter, and subcortical structures (Zollei et al. 2020). Segmentation involves using a multi-atlas approach in which multiple atlases are first registered to subject space and the labels are transferred. The atlases were developed from infant MRI scans (de Macedo Rodrigues et al. 2015). To create the atlases, manually segmented labels were developed using MRI scans from a representative sample of infants (0–2 years of age). In the current study, developmentally appropriate atlases for 4- and 6-month-old infants were employed. The anatomical labels were then fused into a single segmentation result, providing higher accuracy than single-atlas approaches (Iglesias and Sabuncu 2015).

The automatic regional segmentation by the Infant FreeSurfer pipeline was visually qualified on the graphic interface FreeView, which is available with the Freesurfer suite of tools (http://surfer.nmr.mgh.harvard.edu/). Further manual segmentation was employed to correct segmentation errors in the subcortical gray matter using ITK-SNAP (http://www.itksnap.org/). The quantified measurements of the cortical gray, subcortical white matter, and subcortical regions (thalamus, pallidum, putamen, caudate, amygdala, and hippocampus) were extracted. The “brainvol” for global measurements of brain volumes, the “aseg” (Fischl et al. 2002) for the segmentation of subcortical regions, including the basal ganglia (putamen, globus pallidus, caudate, and nucleus accumbens), cerebellum, and brainstem (Desikan et al. 2006). A total of 26 volumes and 204 regionally distributed measurements (regional volume, surface area, and cortical thickness) were extracted from the “aseg” (Fischl et al. 2002) and the “aparc” (Desikan et al. 2006) in each subject, respectively. Segmentation results for representative participants with and without ASD, anatomically annotated (Desikan et al. 2006), are shown in the Fig. S1 in Supplementary Material.

### Statistical analysis

Statistical analyses were performed using Statistical Package for the Social Sciences (v.27 SPSS, Chicago, IL). The main aim of our study is to determine whether responsiveness to RJA and IJA is associated with the differences in subcortical brain volumes which are associated with a later ASD diagnosis in infants with a familial risk for developing ASD. A comprehensive final model was built to address the 2 hypotheses of our aim: to determine (i) whether behavior responsiveness to RJA and IJA are associated with a later diagnosis of ASD in HR infants and (ii) whether volumetric differences in subcortical structures (thalamus, hippocampus, basal ganglia, amygdala, ventral diencephalon, and cerebellum) will be associated with a later ASD diagnosis. First, from all the questionnaires’ items (VABS-II and AOSI), we searched for the keywords related to JA, which included look, eye, watch, point, share, social interest, and attention. Two additional readers reviewed the keywords and agreed that those were the best terms to identify JA behaviors. We selected 11 items from both questionnaires that contained ≥1 keywords. In order to classify the behavioral data from both questionnaires, data reduction methods were applied. A principal component analysis (PCA) was applied to the 11 selected items. The PCA allowed for the creation of new constructs combining data from both questionnaires. Using Varimax rotation, 5 components were extracted (eigenvalues >1). The model was tested for sample adequacy (KMO = 0.493) and for sphericity (Bartlett’s test *P* < 0.001). From the 5 components, 2 composite measures which best defined JA behaviors were chosen from the results of the PCA analysis: RJA and IJA. Subsequently, based on the composites scores, participants were divided into groups: responders to RJA, nonresponders to RJA, responders IJA, and nonresponders to IJA. The RJA composite contained only items from the AOSI, with participants being classified as nonresponders when scores were >0. The IJA composite contained only items from the VABS, and participants receiving behavioral scores of 0 on IJA items were classified as nonresponders.

Data from the nonresponder groups were analyzed using Binomial Logistic Regression. The dependent variable was later ASD diagnosis. The independent variables were cortical (gray and white matter) and subcortical volumes (thalamus, hippocampus, amygdala, nucleus accumbens, ventral diencephalon, and cerebellum), controlling for age, sex, and total cerebral volumes. As we had 2 hypotheses for our aim regarding the predictive ability of volumes and later ASD diagnosis in RJA and IJA nonresponders, alpha level was set to *P* = 0.05/2 or *P* < 0.03 using the Bonferroni correction method.

## Results

### JA composites

The PCA analysis of the behavioral data revealed 5 components, which can be found in Table S1 in Supplementary Material. Two composite scores that best described JA were chosen. Three components that described visual tracking and auditory processing were excluded. The first composite score included items related to eye gaze and shared affect (i.e. eye contact score and social interest and shared affect score), which are aspects related to RJA. The second composite score, IJA, included questions related to pointing and initiating JA (i.e. points to object he or she wants that is out of reach and points or gestures to indicate preference when offered a choice).

#### Responders and nonresponders

From the 2 composites’ scores, IJA and RJA, infants were classified as IJA responders, IJA nonresponders, RJA responders, and RJA nonresponders. 6 participants were excluded from the analysis for the IJA group (*n* = 101) and 11 participants were excluded for the RJA group (*n* = 96) because those infants had completed the behavioral assessments after 9 months of age. Data revealed that for the IJA scores (*n* = 101), the majority of the infants were IJA nonresponders (*n* = 93, 92%), while approximately half of the sample were RJA nonresponders (*n* = 50, 52%).

#### RJA, IJA, brain volume and ASD diagnosis

**Fig. 1:**
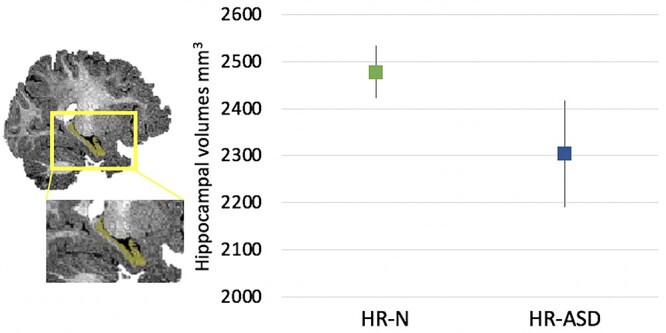
In infants who did not display IJA behaviors, left hippocampal volumes were smaller in high-risk (HR-ASD) infants who later received a diagnosis of ASD compared to high-risk (HR-N) infants who did not receive an ASD diagnosis. Values represent the estimated marginal means of left hippocampal volumes for HR-N and HR-ASD infants, adjusting for biological sex, age, and total cerebral volumes. Estimated marginal means were from a generalized linear model using an identity link function (*P* = 0.006). Errors bars reflect 95% confidence intervals.

Data from the IJA nonresponder group (*n* = 93) were analyzed using a regression method. In the binary logistic regression analysis, subcortical volumes for the thalamus, ventral diencephalon, hippocampus, basal ganglia, and amygdala were entered as predictors in the model, and the ASD diagnosis was used as the outcome variable, controlling for age, sex, and total cerebral volumes. The omnibus test of model coefficient (*P* = 0.049), which is a likelihood-ratio, chi-square test of the model compared to a null model, and the Hosmer and Lemeshow goodness of fit test (*P* = 0.211) demonstrated that our model was appropriate. The results of the regression analysis correctly identified 87.1% of participants who later received an ASD diagnosis. We further tested the classification accuracy of the model using a chi-square test, demonstrating that the classification prediction was better than chance (*X*^2^ = 29.04, *P* < 0.00001, after applying a Yates correction). From the subcortical volumes, the left hippocampus was a significant predictor of ASD diagnosis (*B* = −0.009, aOR = 0.991, *P* = 0.025). The association between hippocampal volumes and ASD diagnosis in the nonresponder IJA group is shown in Fig. 1. The right-thalamus was also a significant predictor of ASD (*B* = −0.016, aOR = 0.984, *P* = 0.026) as well as the left-thalamus (*B* = 0.015, aOR = 1.015, *P* = 0.019). Age was not significant in the model (*P* > 0.05) and sex was borderline (*B* = 2.315, OR = 10.122, *P* = 0.052).

For the RJA nonresponder group, a binomial logistic regression analysis was employed. Subcortical volumes (thalamus, ventral diencephalon, hippocampus, basal ganglia, and amygdala) were predictors in the model, and ASD diagnosis was used as the outcome variable, controlling for age, sex, and total cerebral volumes. No significant associations were evident among the subcortical volumes and ASD diagnosis (all, *P* > 0.03).

## Discussion

In the current study, we examined JA in young infants who had an increased familial likelihood for ASD. We used a data-driven approach to identify the core constructs for IJA and RJA using clinical assessments. We identified that most infants were not responding to IJA, while a larger proportion of infants engaged in RJA. Overall, in the IJA nonresponder group, hippocampal and thalamic volumes were predictive of a later ASD diagnosis, indicating that these brain regions may show an enhanced vulnerability early in life and may be the key predictors of development of ASD. In the RJA nonresponder group, no association between brain volumes and later ASD diagnosis was evident. Findings indicate that the absence of IJA behaviors may be associated with early changes in brain development, which are later associated with an ASD diagnosis.

Atypical JA behaviors have previously been reported in infants who were high risk for developing ASD compared to typically developing infants (Ibanez et al. 2013). Although differences in both IJA and RJA abilities have been observed in previous studies, the absence of IJA responses were more prominent, suggesting that IJA is a better predictor of later ASD diagnosis and symptomatology than RJA (Charman 2003; Ibanez et al. 2013; Gangi et al. 2014). IJA behaviors require not only the ability to follow gaze but also the infant’s motivation to share interest or affect with others (Dawson et al. 2004; Gangi et al. 2014; Mundy 2018). In this sense, IJA requires social information processing in a complex manner, having the infant taking a more active role in IJA than in RJA (Mundy 2018). The infant shifts from being a signal receiver when displaying RJA behaviors to being a signal sender in IJA and motivation to engage in social interactions is required (Mundy 2018). In our study, we found that the majority of infants were IJA nonresponders. In the IJA nonresponder group, thalamus and hippocampal volumes were predictive of the ASD diagnosis, while no association was evident between the brain volumes and diagnosis in the nonresponder RJA group.

Previous literature indicates that infants with a greater likelihood of developing ASD show deficits in communicative and social functioning and in JA (Dawson et al.^2002^; Zwaigenbaum et al. 2005; Ibanez et al. 2013). A study by Ozonoff et al. (2011) reported that only 19% of this population will later be diagnosed with ASD. As IJA requires more skills to be able to process different sources of information (e.g. their own position in space and position of others, direction of gaze, sensory information, and emotional or affective information of others), it is considered to be a more complex ability than RJA (Mundy 2018). In this sense, IJA requires the infant not only to master those skills but also to play an active role in engaging with others. That difference, in terms of the complexity of the behavior, can be hypothesized as a key contributor to the higher percentage of IJA nonresponders compared to RJA.

Impairments in early JA have been identified as one of the earliest indicators of later ASD development (Charman 2003; Bruinsma et al. 2004; Dawson et al. 2004; Werner and Dawson 2005; Ibanez et al. 2013; Bottema-Beutel 2016). Previous studies have found that infants are sensitive to the gaze of others, even from the first days after birth, and prefer faces that engage in mutual gaze (Farroni et al. 2002; Guellai et al. 2020). However, infants and young children later diagnosed with ASD tend to prefer nonsocial stimuli rather than social ones (Pierce et al. 2011; Chawarska et al. 2013; Di Giorgio et al. 2016; Peltola et al. 2018; Gale et al. 2019) and fail to spontaneously orient to the social situation in the environment (Dawson et al. 2004).The failure to orient to social stimuli early in life could possibly lead to impairments in engaging in IJA, and consequently, to later social and communication impairments that are commonly observed in individuals with ASD (Dawson et al. 2004). The lack of early propensity to spontaneously engage in IJA with others might hinder opportunities to build and strengthen brain networks that are necessary for developing social cognition, contributing to a variety of social and language impairments. High-risk infants who do not respond spontaneously to social situations and struggle with early social gaze and JA engagement may be more likely to be diagnosed with ASD.

In our study, infants who were IJA nonresponders and who had smaller thalamic and hippocampal volumes were more likely to later be diagnosed with ASD. The thalamus plays a critical role in the early specialization of the neocortex (Nair et al. 2021). Smaller thalamic volumes have been reported in children diagnosed with ASD compared to control groups (Tamura et al. 2010; Sussman et al. 2015). Evidence from infant and child studies suggests that altered thalamocortical connectivity is associated with ASD symptomatology (Nair et al. 2013, 2015; Chen et al. 2016; Iidaka et al. 2019). Early alterations in the thalamic development and its connectivity have been reported in 6-week-old infants who were at high risk for the development of ASD (Nair et al. 2021). Additionally, in a sample of young children and adolescents with ASD, structural alterations in thalamocortical pathways were associated with social communication impairments along with repetitive behaviors (Nair et al. 2015). Structural alterations in the hippocampus have also been reported in individuals with ASD (Barnea-Goraly et al. 2014; Sussman et al. 2015). The thalamus is a relay center that receives sensory periphery information, such as visual and auditory information, and sends it to the cortex (Nair et al. 2015; Chen et al. 2016; Fu et al. 2019). An infant’s environment presents a variety of sensory information that is processed in their brains. To produce coherent perceptual representations and adequate behavior, this information must be perfectly integrated (Stevenson et al. 2014; Murray et al. 2016). Impairments in sensory function and processing have been observed in individuals with ASD, and the failure to integrate sensory information coming from various sources put those individuals at risk for navigating the social world (Stevenson et al. 2014; Baum et al. 2015). Social and language cues come from different sensory inputs; in turn, integration of multisensory information plays an important role in the social and communication function (Baum et al. 2015). In previous studies, alterations in thalamus-temporal cortex connectivity were associated with language and communication impairments observed in ASD (Chen et al. 2016). The hippocampus is an essential brain region for learning and memory but has also been implicated in shared attention (Nummenmaa and Calder 2009; Anand and Dhikav 2012). In turn, early alterations in the development of these structures in high-risk infants may contribute to impairments with social gaze processing which require active engagement with others.

We report a significant difference in left hippocampal volumes, with smaller volumes predicting a later ASD diagnosis. Atypical hemispheric asymmetry, relating to both structural and functional differences in brain lateralization, is common in ASD (Postema et al. 2019) and are thought to underlie some of the behavioral features characteristic of ASD (Floris et al. 2021), including differences in language abilities (see Pearson and Hodgetts 2020 for a recent review) and motor behaviors (Floris and Howells 2018). While prior research has focused mainly on children and adults, a recent study of high-risk and nominal risk infants and later ASD diagnosis reported no observable differences in functional connectivity among 1-month-old infants, but it did find significant differences when infants were 9 months of age (Rolison et al. 2022). Differences were seen in the extrastriate as well as postcingulate cortex. Connectivity between the postcingulate region and the visual networks are strongly related to IJA behaviors in 1-year-old infants and toddlers (Eggebrecht et al. 2017). Previous work has found an association between the hippocampal connectivity and social difficulties in young children with ASD (Chen et al. 2018). Our right hippocampal finding supports previous reports of atypical hemispheric lateralization in ASD and provides evidence that such differences are visible in the hippocampus in very young infants.

In our study, we found volumetric differences in the thalamus in infants who later developed ASD. The thalamus is a complex brain structure associated with filtering a variety of sensory information, Previous studies have found an increased connectivity between the thalamus and sensory networks in individuals diagnosed with ASD compared to control groups (Fu et al. 2019). Lewis et al. (2017) suggested that alterations in brain networks in ASD individuals are present early in development. It was suggested that the deficits in social communication skills in ASD have a cascade effect due to the deficits in filtering sensory information (Baum et al. 2015; Lewis et al. 2017).

In our study, we found that thalamic volumes predicted ASD diagnosis in high-risk infants who were not responding to IJA. Previous studies have found atypical thalamic connectivity and suggest that this may contribute to impairments in orienting to social information in high-risk infants (Nair et al. 2021). Alterations in thalamic-prefrontal connectivity have been associated with diminished social attention and engagement in high-risk infants, and alterations in thalamic-occipital networks were associated with ASD symptomatology (Nair et al. 2015, 2021). These regions have been associated with the development of social cognition as well as with processing visual information (Nair et al. 2015, 2021). It is possible that our findings related to the alterations in thalamic volumes in high-risk infants underlie the atypical social development in this population and explain why we found that the majority of infants were nonresponders to IJA.

Infants who later develop ASD also fail to orient to social stimuli (Dawson et al. 2004) and one explanation could be rooted in the social motivation theory of ASD (Mundy and Crowson 1997; Dawson et al. 2004; Tomasello et al. 2005). It has been observed that children with ASD lack the coordination to respond to JA but particularly display few IJA behaviors (Tomasello et al. 2005). This major deficit in IJA skills suggests that ASD children may lack the motivation for sharing interests and emotions with others (Tomasello et al. 2005). Social motivation is crucial for IJA, which could explain the greater impairments in IJA rather than in RJA we have reported. It is possible that infants at risk for developing ASD do not experience the social interaction and sharing affect as a reward for continuing to seek interaction throughout their development. These abnormalities in reward neurological systems might explain the failure to attribute reward to social interactions (Dawson et al. 2004; Mundy 2018). Activation of other associated areas of the brain could be associated with motivation and social reward such as the amygdala, the striatum, and the hippocampus (Gordon et al. 2013). Previous studies found that IJA behaviors increase activation in brain areas related to reward, such as the striatum and the hippocampus (Schilbach et al. 2010). Altered hippocampal volumes, as found in our study, may be associated with the atypical reward pathways in the brain. Some individuals with ASD may not process social interactions as rewarding which in turn results in diminished social motivation. This lack of motivation to engage with others could have led to the impairments in IJA observed in our study. The hippocampus might show a greater vulnerability early in life and may be associated with the lack of social motivation in infants who later develop ASD.

Having a sample composed exclusively of infants with autistic siblings, and thus a familial risk of developing ASD, for a longitudinal study is rare, yet further investigation of heterogenous samples is needed to support our findings. As our sample was composed exclusively of infants who had an elevated risk of developing ASD, we cannot presume that the same associations between brain volumes, JA, and later ASD diagnosis exist in typically developing infants who are at low risk for an ASD diagnosis. As IJA is developmentally sensitive and is first exhibited from 2 to 4 months of age, it is possible that these behaviors could not had been completely developed at 4–6 months of age in some participants, which might have impacted our findings. Investigation of the development of JA over longer developmental periods than we were able to include are necessary to determine whether the trajectory of JA behaviors in later infancy (>9 months) show similar associations with that we have reported in the current work.

## Conclusion

In sum, we examined the association between subcortical brain volumes, behavioral measures of JA, and ASD development in at-risk infants. Using a data-driven method, we identified constructs related to both IJA and RJA. We found that the vast majority of infants in our sample were nonresponders to IJA, and from this group, we found that hippocampal and thalamic volumes predicted later ASD diagnosis. These findings suggest that these brain regions may have enhanced vulnerability early in life and may be key predictors of ASD development in infants who are at high risk. A better understanding of the early signs of social gaze and JA, as well as the neural mechanisms behind those behaviors, could help identify targets for intervention as well as biomarkers to promote improved social and behavioral outcomes in infants who are at high risk for the development of ASD.

## Supplementary Material

Supplementary_Information_tgac029

## References

[ref1] Adamson LB , Bakeman R, Suma K, Robins DL. An expanded view of joint attention: skill, engagement, and language in typical development and autism. Child Dev. 2019:90(1):e1–e18. 10.1111/cdev.12973. 28991358 PMC5891390

[ref2] American Psychiatric Association , editors. Diagnostic and statistical manual of mental disorders: DSM-5. 5th ed. Washington, D.C.: American Psychiatric Association; 2013

[ref3] Anand KS , Dhikav V. Hippocampus in health and disease: an overview. Ann Indian Acad Neurol. 2012:15(4):239–246. 10.4103/0972-2327.104323. 23349586 PMC3548359

[ref67] Avino, T.A., Barger, N., Vargas, M.V., Carlson, E.L., Amaral, D.G., Bauman, M.D. and Schumann, C.M. Neuron numbers increase in the human amygdala from birth to adulthood, but not in autism. PNAS. 2018:115(14):3710–3715. 29559529 10.1073/pnas.1801912115 PMC5889677

[ref4] Barnea-Goraly N , Frazier TW, Piacenza L, Minshew NJ, Keshavan MS, Reiss AL, Hardan AY. A preliminary longitudinal volumetric MRI study of amygdala and hippocampal volumes in autism. Prog Neuro-Psychopharmacol Biol Psychiatry. 2014:48:124–128. 10.1016/j.pnpbp.2013.09.010. PMC8655120 24075822

[ref5] Baum SH , Stevenson RA, Wallace MT. Behavioral, perceptual, and neural alterations in sensory and multisensory function in autism spectrum disorder. Prog Neurobiol. 2015:134:140–160. 10.1016/j.pneurobio.2015.09.007. 26455789 PMC4730891

[ref83] Bigler ED , Tate DF, Neeley ES, Wolfson LJ, Miller MJ, Rice SA, Cleavinger H, Anderson C, Coon H, Ozonoff S, Johnson M, Dinh E, Lu J, Mc Mahon W, Lainhart JE. Temporal lobe, autism, and macrocephaly. AJNR Am J Neuroradiol. 2003:24(10):2066–76. 14625235 PMC8148899

[ref7] Bottema-Beutel K . Associations between joint attention and language in autism spectrum disorder and typical development: a systematic review and meta-regression analysis. Autism Res. 2016:9(10):1021–1035. 10.1002/aur.1624. 27059941

[ref8] Bruinsma Y , Koegel RL, Koegel LK. Joint attention and children with autism: a review of the literature. Ment Retard Dev Disabil Res Rev. 2004:10(3):169–175. 10.1002/mrdd.20036. 15611988

[ref10] Bryson SE , Zwaigenbaum L, McDermott C, Rombough V, Brian J. The autism observation scale for infants: scale development and reliability data. J Autism Dev Disord. 2008:38:731–738. 10.1007/s10803-007-0440-y. 17874180

[ref79] Bryson, S.E. and Zwaigenbaum, L. Autism observation scale for infants. Comprehensive guide to autism. New York: Springer, 2014, 299–310.

[ref12] Charman T . Why is joint attention a pivotal skill in autism? Philos Trans R Soc Lond Ser B Biol Sci. 2003:358:315–324. 10.1098/rstb.2002.1199. 12639329 PMC1693124

[ref13] Chawarska K , Macari S, Shic F. Decreased spontaneous attention to social scenes in 6-month-old infants later diagnosed with autism spectrum disorders. Biol Psychiatry. 2013:74(3):195–203. 10.1016/j.biopsych.2012.11.022. 23313640 PMC3646074

[ref14] Chen H , Uddin LQ, Zhang Y, Duan X, Chen H. Atypical effective connectivity of thalamo-cortical circuits in autism spectrum disorder. Autism Res. 2016:9(11):1183–1190. 10.1002/aur.1614. 27868393

[ref85] Chen H , Wang J, Uddin LQ, Wang X, Guo X, Lu F, Duan X, Wu L, Chen H. Aberrant functional connectivity of neural circuits associated with social and sensorimotor deficits in young children with autism spectrum disorder. Autism Res. 2018:11(12):1643–1652. 10.1002/aur.2029 30475453 10.1002/aur.2029 PMC6281874

[ref80] Community-University Partnership for the Study of Children, Youth, and Families . 2011. Review of the Vineland Adaptive Behavior Scales-Second Edition (Vineland-II). Edmonton, Alberta, Canada.

[ref16] Dawson G , Webb S, Schellenberg GD, Dager S, Friedman S, Aylward E, Richards T. Defining the broader phenotype of autism: genetic, brain, and behavioral perspectives. Dev Psychopathol. 2002:14:581–611. 10.1017/S0954579402003103. 12349875

[ref17] Dawson G , Toth K, Abbott R, Osterling J, Munson J, Estes A, Liaw J. Early social attention impairments in autism: social orienting, joint attention, and attention to distress. Dev Psychol. 2004:40(2):271–283. 10.1037/0012-1649.40.2.271. 14979766

[ref81] de Macedo Rodrigues, K., Ben-Avi, E., Sliva, D.D., Choe, M.S., Drottar, M., Wang, R., Fischl, B., Grant, P.E. and Zöllei, L. A FreeSurfer-compliant consistent manual segmentation of infant brains spanning the 0–2 year age range. Front Hum Neurosci. 2015:9:21. 25741260 10.3389/fnhum.2015.00021 PMC4332305

[ref18] Desikan RS , Segonne F, Fischl B, Quinn BT, Dickerson BC, Blacker D, Killiany RJ. An automated labeling system for subdividing the frontiers in human cerebral cortex on MRI scans into gyral based regions of interest. NeuroImage. 2006:31(3):968–980. 10.1016/j.neuroimage.2006.01.021. 16530430

[ref64] D’Entremont, B. A perceptual–attentional explanation of gaze following in 3‐and 6‐month‐olds. Dev Sci. 2000:3(3):302–311.

[ref19] Di Giorgio E , Frasnelli E, Rosa Salva O, Scattoni ML, Puopolo M, Tosoni D, Vallortigara G. Difference in visual social predispositions between newborns at low- and high-risk for autism. Sci Rep. 2016:6:26395. 10.1038/srep26395. 27198160 PMC4873740

[ref78] Eggebrecht, A.T., Elison, J.T., Feczko, E., Todorov, A., Wolff, J.J., Kandala, S., Adams, C.M., Snyder, A.Z., Lewis, J.D., Estes, A.M. and Zwaigenbaum, L. Joint attention and brain functional connectivity in infants and toddlers. Cereb Cortex. 2017:27(3):1709–1720. 28062515 10.1093/cercor/bhw403 PMC5452276

[ref20] Emery NJ . The eyes have it: the neuroethology, function and evolution of social gaze. Neurosci Biobehav Rev. 2000:24:581–604. 10940436 10.1016/s0149-7634(00)00025-7

[ref21] Farroni T , Csibra G, Simion F, Johnson MH. Eye contact detection in humans from birth. PNAS. 2002:99(14):9602–9605. https://www.pnas.org/doi/full/10.1073/pnas.152159999. 12082186 10.1073/pnas.152159999 PMC123187

[ref22] Fischl B . FreeSurfer. NeuroImage. 2012:62(2):774–781. 10.1016/j.neuroimage.2012.01.021. 22248573 PMC3685476

[ref23] Fischl B , Salat DH, Busa E, Albert M, Dieterich M, Haselgrove C, Van der Kouwe A, Killiany R, Kennedy K, Klaveness S, et al. Whole brain segmentation: automated labeling of neuroanatomical structures in the human brain. Neuron. 2002:33:341–355. 11832223 10.1016/s0896-6273(02)00569-x

[ref76] Floris, D.L. and Howells, H. Atypical structural and functional motor networks in autism. Prog Brain Res. 2018:238:207–248. 30097193 10.1016/bs.pbr.2018.06.010

[ref84] Floris DL , Filho JOA, Lai MC, Giavasis S, Oldehinkel M, Mennes M, Charman T, Tillmann J, Dumas G, Ecker C, Dell'Acqua F, Banaschewski T, Moessnang C, Baron-Cohen S, Durston S, Loth E, Murphy DGM, Buitelaar JK, Beckmann CF, Milham MP, Di Martino A. Towards robust and replicable sex differences in the intrinsic brain function of autism. Mol Autism. 2021:12(1):19. 10.1186/s13229-021-00415-z. PMC7923310 33648569

[ref24] Fu Z , Tu Y, Di X, Du Y, Sui J, Biswal BB, Calhoun VD. Transient increased thalamic-sensory connectivity and decreased whole-brain dynamism in autism. NeuroImage. 2019:190:191–204. 10.1016/j.neuroimage.2018.06.003. 29883735 PMC6281849

[ref25] Gaffrey MS , Markert S, Yu C. Social origins of self-regulated attention during infancy and their disruption in autism spectrum disorder: implications for early intervention. Dev Psychopathol. 2020:32(4):1362–1374. 10.1017/S0954579420000796. 32693862 PMC7670885

[ref26] Gale CM , Eikeseth S, Klintwall L. Children with autism show atypical preference for non-social stimuli. Sci Rep. 2019:9:10355. 10.1038/s41598-019-46705-8. 31316161 PMC6637109

[ref27] Gangi DN , Ibanez LV, Messinger DS. Joint attention initiation with and without positive affect: risk group differences and associations with ASD symptoms. J Autism Dev Disord. 2014:44:1414–1424. 10.1007/s10803-013-2002-9. 24281421 PMC4024338

[ref28] Gong T , Shuai L. Modelling the coevolution of joint attention and language. Proc Biol Sci. 2012:279:4643–4651. 10.1098/rspb.2012.1431. 22977146 PMC3479722

[ref29] Gordon I , Eilbott JA, Feldman R, Pelphrey KA, Vander Wyk BC. Social, reward, and attention brain networks are involved when online bids for joint attention are met with congruent versus incongruent responses. Soc Neurosci. 2013:8(6):544–554. 10.1080/17470919.2013.832374. 24044427

[ref30] Gredebäck G , Fikke L, Melinder A. The development of joint visual attention: a longitudinal study of gaze following during interactions with mothers and strangers. Dev Sci. 2010:13(6):839–848. 10.1111/j.1467-7687.2009.00945.x. 20977555

[ref31] Guellai B , Hausberger M, Chopin A, Streri A. Premises of social cognition: newborns are sensitive to a direct versus a faraway gaze. Sci Rep. 2020:10:9796. 10.1038/s41598-020-66576-8. 32555228 PMC7299991

[ref32] Haith MM , Bergman T, Moore MJ. Eye contact and face scanning in early infancy. Science. 1977:198(4319):853–855 https://www.jstor.org/stable/1745434. 918670 10.1126/science.918670

[ref33] Ibanez LV , Grantz CJ, Messinger DS. The development of referential communication and autism symptomatology in high-risk infants. Infancy. 2013:18(5): 687–707. 10.1111/j.1532-7078.2012.00142.x. PMC3880657 24403864

[ref73] Iglesias, J.E. and Sabuncu, M.R. Multi-atlas segmentation of biomedical images: a survey. Med Image Anal. 2015:24(1):205–219. 26201875 10.1016/j.media.2015.06.012 PMC4532640

[ref34] Iidaka T , Kogata T, Mano Y, Komeda H. Thalamocortical hyperconnectivity and amygdala-cortical hypoconnectivity in male patients with autism spectrum disorder. Front Psychiatry. 2019:10:252. 10.3389/fpsyt.2019.00252. 31057443 PMC6482335

[ref35] Lewis JD , Evans AC, Pruett JR Jr, Botteron KN, McKinstry RC, Zwaigenbaum L, Estes, A.M., Collins, D.L., Kostopoulos, P., Gerig, G. and Dager, S.R., 2017. Infant Brain Imaging Study, N. The emergence of network inefficiencies in infants with autism spectrum disorder. Biol Psychiatry. 2017:82(3):176–185. 10.1016/j.biopsych.2017.03.006. 28460842 PMC5524449

[ref68] Li, G., Chen, M.H., Li, G., Wu, D., Lian, C., Sun, Q., Shen, D. and Wang, L. A longitudinal MRI study of amygdala and hippocampal subfields for infants with risk of autism. In International Workshop on Graph Learning in Medical Imaging. 2019, 164–171. Springer, Cham. 10.1007/978-3-030-35817-4_20 PMC7043018 32104792

[ref72] Li, C., Du, W., Liu, H., Yang, M., Xu, H., Wu, J. and Wang, Z. A hippocampus-inspired illumination time-resolved device for neural coding. Sci China Mater. 2022:65(4):1087–1093.

[ref36] Lord C , Risi S, Lambrecht L, Cook J, Edwin H, Leventhal BL, Dilavore PC, Rutter M. The Autism Diagnostic Observation Schedule—Generic: A Standard Measure of Social and Communication Deficits Associated with the Spectrum of Autism. J Autism Dev Disord. 2000:30:205–223. 10.1023/a:1005592401947. 11055457

[ref38] Mosconi MW , Cody-Hazlett H, Poe MD, Gerig G, Gimpel-Smith R, Piven J. Longitudinal study of amygdala volume and joint attention in 2- to 4-year-old children with autism. Arch Gen Psychiatry. 2009:66(5):509–516. 10.1001/archgenpsychiatry.2009.19. 19414710 PMC3156446

[ref39] Mundy P . A review of joint attention and social-cognitive brain systems in typical development and autism spectrum disorder. Eur J Neurosci. 2018:47(6):497–514. 10.1111/ejn.13720. 28922520

[ref40] Mundy P , Crowson M. Joint attention and early social communication: implications for research on intervention with autism. J Autism Dev Disord. 1997:27(6):653–676 https://www.ncbi.nlm.nih.gov/pubmed/9455727. 9455727 10.1023/a:1025802832021

[ref41] Mundy P , Jarrold W. Infant joint attention, neural networks and social cognition. Neural Netw. 2010:23(8-9):985–997. 10.1016/j.neunet.2010.08.009. 20884172 PMC2963105

[ref42] Murray MM , Lewkowicz DJ, Amedi A, Wallace MT. Multisensory processes: a balancing act across the lifespan. Trends Neurosci. 2016:39(8):567–579. 10.1016/j.tins.2016.05.003. 27282408 PMC4967384

[ref43] Nair A , Treiber JM, Shukla DK, Shih P, Muller RA. Impaired thalamocortical connectivity in autism spectrum disorder: a study of functional and anatomical connectivity. Brain. 2013:136(6):1942–1955. 10.1093/brain/awt079. 23739917 PMC3673456

[ref44] Nair A , Carper RA, Abbott AE, Chen CP, Solders S, Nakutin S, Muller RA. Regional specificity of aberrant thalamocortical connectivity in autism. Hum Brain Mapp. 2015:36:4497–4511. 10.1002/hbm.22938. 26493162 PMC4768761

[ref45] Nair A , Jalal R, Liu J, Tsang T, McDonald NM, Jackson L, Dapretto M. Altered thalamocortical connectivity in 6-week-old infants at high familial risk for autism spectrum disorder. Cereb Cortex. 2021:31(9):4191–4205. 10.1093/cercor/bhab078. 33866373 PMC8328203

[ref82] Nicolson R , DeVito TJ, Vidal CN, Sui Y, Hayashi KM, Drost DJ, Williamson PC, Rajakumar N, Toga AW, Thompson PM. Detection and mapping of hippocampal abnormalities in autism. Psychiatry Res. 2006:148(1):11–21. 10.1016/j.pscychresns.2006.02.005. Epub 2006 Oct 23. PMID: 17056234. 17056234 10.1016/j.pscychresns.2006.02.005

[ref66] Nordahl, C.W., Scholz, R., Yang, X., Buonocore, M.H., Simon, T., Rogers, S. and Amaral, D.G. Increased rate of amygdala growth in children aged 2 to 4 years with autism spectrum disorders: a longitudinal study. Archives of general psychiatry. 2012:69(1):53–61. 22213789 10.1001/archgenpsychiatry.2011.145 PMC3632313

[ref46] Nummenmaa L , Calder AJ. Neural mechanisms of social attention. Trends Cogn Sci. 2009:13(3):135–143. 10.1016/j.tics.2008.12.006. 19223221

[ref47] Ofner M , Coles A, Decou ML, Do MT, Bienek A, Snider J, Ugnat AM. Autism spectrum disorder among children and youth in Canada 2018: a report of the National Autism Spectrum Disorder Surveillance System. 1st ed. Ottawa: Public Health Agency of Canada; 2018

[ref48] Ozonoff S , Young GS, Carter A, Messinger D, Yirmiya N, Zwaigenbaum L, Stone WL. Recurrence risk for autism spectrum disorders: a Baby Siblings Research Consortium study. Pediatrics. 2011:128(3):e488–e495. 10.1542/peds.2010-2825. 21844053 PMC3164092

[ref49] Payakachat N , Tilford JM, Ungar WJ. National Database for Autism Research NDAR: big data opportunities for health services research and health technology assessment. PharmacoEconomics. 2016:34:127–138. 10.1007/s40273-015-0331-6. 26446859 PMC4761298

[ref75] Pearson, A. and Hodgetts, S. Can cerebral lateralisation explain heterogeneity in language and increased non-right handedness in autism? A literature review. Res Dev Disabil. 2020:105:103738. 10.1016/j.ridd.2020.103738 32721786

[ref50] Peltola MJ , Yrttiaho S, Leppanen JM. Infants' attention bias to faces as an early marker of social development. Dev Sci. 2018:21(6):e12687. 10.1111/desc.12687. 29971869

[ref51] Pierce K , Conant D, Hazin R, Stoner R, Desmond J. Preference for geometric patterns early in life as a risk factor for autism. Arch Gen Psychiatry. 2011:68(1):101–109. 10.1001/archgenpsychiatry.2010.113. 20819977 PMC4894313

[ref74] Postema, M.C., Van Rooij, D., Anagnostou, E., Arango, C., Auzias, G., Behrmann, M., Calderoni, S., Calvo, R., Daly, E., Deruelle, C. and Di Martino, A. Altered structural brain asymmetry in autism spectrum disorder in a study of 54 datasets. Nat Commun. 2019:10(1):1–12. 31673008 10.1038/s41467-019-13005-8 PMC6823355

[ref77] Rolison, M., Lacadie, C., Chawarska, K., Spann, M. and Scheinost, D. Atypical intrinsic hemispheric interaction associated with autism spectrum disorder is present within the first year of life. Cereb Cortex. 2022:32(6):1212–1222. 34424949 10.1093/cercor/bhab284 PMC8924430

[ref52] Schilbach L , Wilms M, Eickhoff SB, Romanzetti S, Tepest R, Bente GJ, Shah NJ, Fink GR, Vogeley K. Minds made for sharing: initiating joint attention recruits reward-related neurocircuitry. J Cogn Neurosci. 2010:22(12):2702–2715. 19929761 10.1162/jocn.2009.21401

[ref70] Schuetze, M., Park, M.T.M., Cho, I.Y., MacMaster, F.P., Chakravarty, M.M. and Bray, S.L. Morphological alterations in the thalamus, striatum, and pallidum in autism spectrum disorder. Neuropsychopharmacology. 2016:41(11):2627–2637. 27125303 10.1038/npp.2016.64 PMC5026732

[ref71] Schumann, C.M., Hamstra, J., Goodlin-Jones, B.L., Lotspeich, L.J., Kwon, H., Buonocore, M.H., Lammers, C.R., Reiss, A.L. and Amaral, D.G. The amygdala is enlarged in children but not adolescents with autism; the hippocampus is enlarged at all ages. J Neurosci Res. 2004:24(28):6392–6401. 10.1523/JNEUROSCI.1297-04.2004 PMC6729537 15254095

[ref69] Seguin, D., Pac, S., Wang, J., Nicolson, R., Martinez‐Trujillo, J. and Duerden, E.G. Amygdala subnuclei development in adolescents with autism spectrum disorder: Association with social communication and repetitive behaviors. Brain Behav. 2021:11(8):e2299. 34333868 10.1002/brb3.2299 PMC8413788

[ref53] Sparrow SS . Vineland adaptive behavior scales. Encyclopedia of Clinical Neuropsychologyp. 2011:2618–2621. Springer, New York, NY. 10.1007/978-0-387-79948-3_1602.

[ref54] Stevenson RA , Siemann JK, Woynaroski TG, Schneider BC, Eberly HE, Camarata SM, Wallace MT. Evidence for diminished multisensory integration in autism spectrum disorders. J Autism Dev Disord. 2014:44:3161–3167. 10.1007/s10803-014-2179-6. 25022248 PMC4224676

[ref55] Sussman D , Leung RC, Vogan VM, Lee W, Trelle S, Lin S, Taylor MJ. The autism puzzle: diffuse but not pervasive neuroanatomical abnormalities in children with ASD. Neuroimage Clin. 2015:8:170–179. 10.1016/j.nicl.2015.04.008. 26106541 PMC4473820

[ref56] Swanson MR , Serlin GC, Siller M. Broad autism phenotype in typically developing children predicts performance on an eye-tracking measure of joint attention. J Autism Dev Disord. 2013:43:707–718. 10.1007/s10803-012-1616-7. 22847297 PMC4369790

[ref57] Tamura R , Kitamura H, Endo T, Hasegawa N, Someya T. Reduced thalamic volume observed across different subgroups of autism spectrum disorders. Psychiatry Res. 2010:184(3):186–188. 10.1016/j.pscychresns.2010.07.001. 20850279

[ref58] Tomasello M , Carpenter M, Call J, Behne T, Moll H. Understanding and sharing intentions: the origins of cultural cognition. Behav Brain Sci. 2005:28(5):675–691. 10.1017/S0140525X05000129. 16262930

[ref65] Van Rooij, D., Anagnostou, E., Arango, C., Auzias, G., Behrmann, M., Busatto, G.F., Calderoni, S., Daly, E., Deruelle, C., Di Martino, A. and Dinstein, I. Cortical and subcortical brain morphometry differences between patients with autism spectrum disorder and healthy individuals across the lifespan: results from the ENIGMA ASD Working Group. Am J Psychiatry. 2018:175(4):359–369. 29145754 10.1176/appi.ajp.2017.17010100 PMC6546164

[ref60] Werner E , Dawson G. Validation of the phenomenon of autistic regression using home videotapes. Arch Gen Psychiatry. 2005:62:889–895. 16061766 10.1001/archpsyc.62.8.889

[ref61] Zollei L , Iglesias JE, Ou Y, Grant PE, Fischl B. Infant FreeSurfer: an automated segmentation and surface extraction pipeline for T1-weighted neuroimaging data of infants 0-2 years. NeuroImage. 2020:218:116946. 10.1016/j.neuroimage.2020.116946. 32442637 PMC7415702

[ref62] Zwaigenbaum L . Advances in the early detection of autism. Curr Opin Neurol. 2010:23(2):97–102. 10.1097/WCO.0b013e3283372430. 20154615

[ref63] Zwaigenbaum L , Bryson S, Rogers T, Roberts W, Brian J, Szatmari P. Behavioral manifestations of autism in the first year of life. Int J Dev Neurosci. 2005:23(2-3):143–152. 10.1016/j.ijdevneu.2004.05.001. 15749241

