# Supplementary material for: Joint attention in infants at high familial risk for autism spectrum disorder and the association with thalamic and hippocampal macrostructure
Source: Cereb Cortex Commun. 2022 Jul 22;3(3):tgac029. doi: 10.1093/texcom/tgac029 (PMC9441013; doi:10.1093/texcom/tgac029)
Supplement: Supplementary_Information_tgac029 [file supplementary_information_tgac029.docx]

Supplementary Information

Table S1. Principal components analysis of gaze data

|  |  | **Eigenvalues** | | |
| --- | --- | --- | --- | --- |
|  | **Questions** | **Total** | **% of Variance** | **Cumulative %** |
| **Component 1** | Eye contact score | 1.977 | 17.969 | 17.969 |
|  | Social interest and shared affect score |  |  |  |
| **Component 2** | Looks at face of parent or caregiver. | 1.811 | 16.463 | 34.432 |
|  | Watches (that is, follows with eyes) someone moving by crib or bed for 5 seconds or more. |  |  |  |
| **Component 3** | Turns eyes and head toward sound. | 1.708 | 15.526 | 49.959 |
|  | Looks toward parent or caregiver when hearing parent's or caregiver's voice. |  |  |  |
| **Component 4** | Points to object he or she wants that is out of reach. | 1.52 | 13.82 | 63.779 |
|  | Points or gestures to indicate preference when offered a choice (for example: "Do you want this one or that one?" etc.) |  |  |  |
| **Component 5** | Points to common objects in a book or magazine as they are named (for example, dog, car, cup, key, etc.). | 1.085 | 9.868 | 73.646 |
|  | Makes sounds or gestures (for example, waves arms) to get parent's or caregivers’ attention. |  |  |  |

Principal components analysis applied to questionnaire items from the Vineland Adaptive Behavior Scales, 2^nd^ edition (VABS-II) and the Autism Observation Scale for Infants (AOSI). Questionnaire items from component 1 were used to create the responding to joint attention grouping variable (RJA). Questionnaire items from component 4 were used for the initiating joint attention grouping variable (IJA).


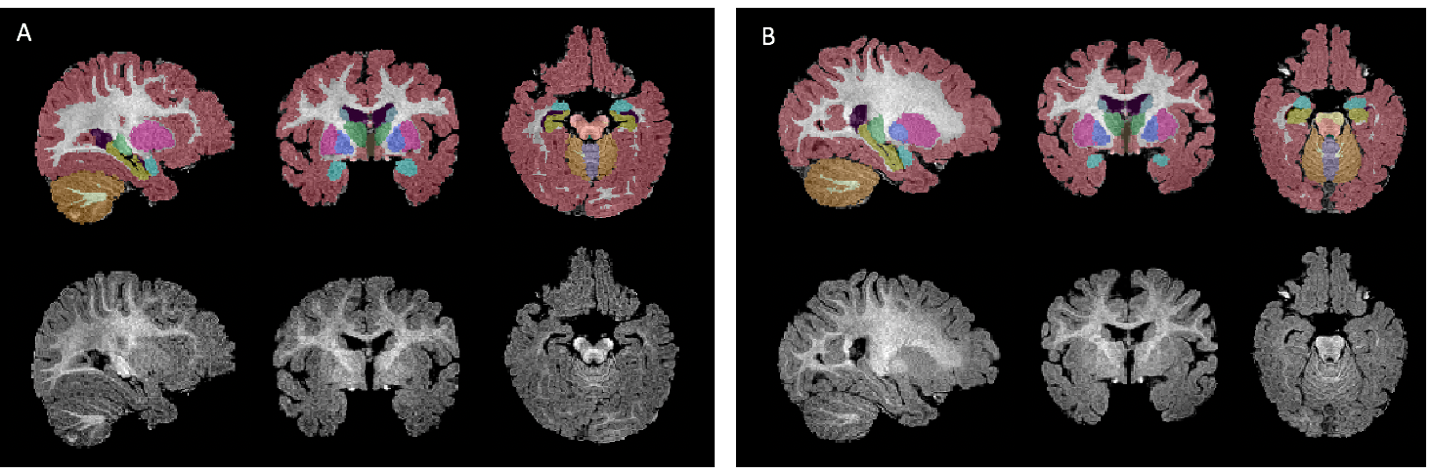


Figure S1. Post-processed T1-weighted MRI scans that segmented using infant Freesurfer in (A) a high risk infant who was later diagnosed with ASD and (B) a high risk infant who did not receive an ASD diagnosis.
